# Underuse of Antiviral Drugs to Prevent Progression to Severe COVID-19 — Veterans Health Administration, March–September 2022

**DOI:** 10.15585/mmwr.mm7303a2

**Published:** 2024-01-25

**Authors:** Paul A. Monach, Sonia T. Anand, Nathanael R. Fillmore, Jennifer La, Westyn Branch-Elliman

**Affiliations:** ^1^VA Boston Cooperative Studies Program, Boston, Massachusetts; ^2^Department of Medicine, VA Boston Healthcare System, Boston, Massachusetts; ^3^Harvard Medical School, Boston, Massachusetts; ^4^Dana-Farber Cancer Institute, Boston, Massachusetts; ^5^VA Boston Center for Healthcare Organization & Implementation Research, Boston, Massachusetts.

**Summary**

**What is already known about this topic?**

Antiviral drugs reduce progression to severe COVID-19 among high-risk patients with nonsevere disease; however, reported use is low.

**What is added by this report?**

Review of 110 immunosuppressed patients with nonsevere COVID-19 at risk for progression who did not receive an antiviral drug found that 80% were not offered such treatment. For nearly one half of these, the only reason given for not offering antiviral treatment was mild symptoms. Other reasons included symptom duration >5 days (22.7%), lack of symptoms (22.7%), and concern about drug interactions (5.7%). One fifth of the 110 patients were offered treatment but declined.

**What are the implications for public health practice?**

Education of providers, patients, and staff members tasked with follow-up calls might increase use of antiviral medications for mild-to-moderate COVID-19, especially if combined with advance planning for possible antiviral treatment at the time of testing or earlier.

## Abstract

Antiviral drugs reduce the rate of progression to severe COVID-19 when given to patients with mild-to-moderate disease within 5 days of symptom onset. Despite being recommended for patients at high risk for progression to severe COVID-19 because of age or chronic conditions, reported antiviral use among the general adult population has been ≤35%. To ascertain reasons for underuse of antiviral medications to prevent severe COVID-19 and propose interventions accordingly, a detailed review was conducted of 110 Veterans Health Administration patients with mild-to-moderate infection at high risk for progression because of underlying conditions (organ transplantation or hematologic malignancies) who did not receive an antiviral drug. Among these 110 patients, all of whom had received COVID-19 vaccine, 22 (20.0%) were offered treatment but declined, and 88 (80.0%) were not offered treatment. Among the 88 patients not offered treatment, provider reasons included symptom duration of >5 days (22.7%), concern about possible drug interactions (5.7%), or absence of symptoms (22.7%); however, among nearly one half (43 of 88; 48.9%) of these patients, no reason other than mild symptoms was given. Among 24 (55.8%) of those 43 patients, follow-up was limited to telephone calls to report test results and inquire about symptom evolution, with no documentation of treatment being offered. These findings suggest that education of patients, providers, and medical personnel tasked with follow-up calls, combined with advance planning in the event of a positive test result, might improve the rate of recommended antiviral medication use to prevent severe COVID-19–associated illness, including death.

## Introduction

Use of the antiviral drugs nirmatrelvir/ritonavir (Paxlovid) and remdesivir (Veklury) is approved by the Food and Drug Administration (FDA); molnupiravir (Lagevrio) is authorized for emergency use.* These antiviral medications reduce the risk of hospitalization and death and are recommended for patients with mild-to-moderate COVID-19 who are at high risk for progression to severe COVID-19 because of age or medical conditions (*1*). All three of these drugs have retained activity against different circulating SARS-CoV-2 variants. In contrast, by late 2022, monoclonal antibodies had lost activity against prevalent variants (*2*).

Despite demonstrated effectiveness and guideline endorsement (*3*,*4*), use of antiviral medications appears to be considerably lower than expected, based on the prevalence of risk factors for severe COVID-19. A recent study of patients in the Veterans Health Administration (VA) reported use of outpatient antiviral medications among 24% of all documented SARS-CoV-2 infections in 2022, remaining at that level through early 2023 (*5*). Description of the untreated comparison group clearly showed that many patients would have met treatment criteria. Similar overall rates of use (maximum = 34%) were observed in a large cohort from health care systems participating in the National Patient-Centered Clinical Research Network (PCORnet) (*6*).

To identify barriers to antiviral use that might be addressed through novel implementation strategies, a sample of VA patients with COVID-19 was reviewed to ascertain the reasons for the nontreatment of patients with mild-to-moderate disease at the time of initial evaluation and testing. These patients had all received COVID-19 vaccination and had one of three relatively common conditions associated with severe immunocompromise, placing them at risk for progression to severe COVID-19 despite vaccination (solid organ transplantation, chronic lymphocytic leukemia [CLL], or plasma cell malignancies).

## Methods

### Study Cohort and Patient Characteristics

Data on vaccination and SARS-CoV-2 infection and demographic and clinical data were obtained from the VA COVID-19 Shared Data Resource (*7*) and from electronic medical records (EMRs) and vital status in the Corporate Data Warehouse, respectively. The VA Joint Legacy Viewer interface, a web application that provides an integrated read-only view of EMR data from the VA, Department of Defense, and community partners^†^^,^^§^ was used for chart review.

The first date of a documented positive SARS-CoV-2 test result was used to define infection after vaccination. Data were limited to infections documented from March 1, 2022 (when effective oral antivirals became widely available to treat outpatients with mild-to-moderate COVID-19) (*5*), through September 30, 2022. Patients with a diagnosis of solid organ transplantation, CLL, or plasma cell malignancies were initially identified by single use of *International Classification of Diseases, Tenth Revision* (ICD-10) codes during January 1, 2021–September 30, 2022. Use of immunosuppressive or antineoplastic drugs was ascertained 3–12 months before the date of infection (depending on the drug). Nonuse of oral (by outpatient pharmacy dispensing records) or intravenous antiviral drugs (by orders placed) was preliminarily ascertained from 5 days before (to identify treatment based on a non-VA test) to 28 days after the VA test. The electronic search for prescription of an antiviral was limited to nirmatrelvir/ritonavir, molnupiravir, and monoclonal antibodies, because pharmacy records preclude distinguishing between remdesivir use to treat versus prevent severe COVID-19 (*5*). Severe COVID-19 was defined as 1) death within 28 days after the positive test result, or 2) hospitalization with either use of dexamethasone or evidence of at least mild hypoxemia (minimum oxygen saturation <94% or any use of supplemental oxygen) (*8*).

Comorbidities were defined using ICD-10 codes per the Chronic Conditions Warehouse^¶^ during the 12 months preceding initial vaccination.** Presence of comorbidities was summarized as a count (0–6) of six chronic conditions that have been associated with severe COVID-19 after vaccination (*8*,*9*). To improve representativeness of the high-risk VA population and to reduce the prevalence of missing data, the study was limited to patients who had documentation of either 2 doses of an mRNA COVID-19 vaccine or 1 dose of an adenoviral COVID-19 vaccine.

### Sampling and Inclusion Criteria

The full cohort comprised 1,196 VA patients who received a vaccination for COVID-19; had an ICD-10 code indicating solid organ transplantation, CLL, or plasma cell malignancy; and had received a positive SARS-CoV-2 test result during March 1–September 30, 2022. A random sample of patients without EMR evidence of prescription of antiviral medication was then selected for detailed chart review. To obtain a random sample, a random number was assigned to each case, and cases were reviewed in sequential order beginning with the lowest assigned number until the target number of cases meeting criteria for inclusion was obtained.

After chart review, patients were included in the analysis of reasons for nonreceipt of antiviral medication if the patients met the following criteria: 1) confirmation of a diagnosis of solid organ transplantation, CLL, or plasma cell malignancy; 2) confirmation of no evidence of antiviral use at either a VA or non-VA facility; and 3) initial medical evaluation of mild-to-moderate COVID-19. Review proceeded until 110 cases meeting inclusion criteria were classified. A target of 100 cases was chosen to allow reasonable precision in estimating the frequency of a common event (for example, an outcome of 28% would have a 95% CI of 20%–36%) for a time-intensive process. The aim was to include similar numbers of cases of solid organ transplantation and hematologic malignancies (including numbers of patient with CLL or plasma cell malignancies). 

### Data Analysis

A member of the study team conducted chart review and classified each case using the following criteria: 1) antiviral was offered, and the patient declined treatment; or antiviral was not offered because of 2) symptom duration >5 days; 3) concern about drug interactions expressed by the provider; 4) administrative barriers or delays; or 5) not determined to be indicated because of mild or asymptomatic disease, with the option for antiviral treatment either rejected by the provider or not mentioned. Reference to a patient’s or provider’s concern for Paxlovid rebound (a phenomenon of temporary recurrence of symptoms shortly after completing a standard 5-day Paxlovid treatment) was also recorded.

Among cases that were reviewed but did not meet inclusion criteria for analysis, the proportion for which an antiviral was received for mild-to-moderate COVID-19 but was not discernible from the initial electronic screen of EMR (e.g., remdesivir given for 3 days, or an oral antiviral given in the emergency department or through a non-VA pharmacy) was determined, to better estimate under usage of antivirals. This activity was determined to be exempt from Institutional Review Board (IRB) oversight or a requirement for informed consent by the IRB of the VA Boston Healthcare System.^††^

## Results

### Patient Characteristics

Among all 1,196 patients, 96% were male, nearly two thirds (64%) were non-Hispanic White, and 84% had received a COVID-19 vaccine booster dose (Table 1). Those with solid organ transplantation (317) were younger (mean age = 64.6 years) than were the 472 patients with CLL (75.3 years) or the 407 patients with plasma cell malignancies (72.7 years). All patients with solid organ transplantation and most patients with CLL (67.4%) or plasma cell malignancies (81.8%) were currently receiving immunosuppressive or antineoplastic medications (Supplementary Table; https://stacks.cdc.gov/view/cdc/141955). Overall, 861 (72.0%) patients were initially classified in EMR as not having received antiviral drugs, including 223 (70.3%) with solid organ transplantation, 324 (68.6%) with CLL, and 314 (77.1%) with plasma cell malignancies. Among the 207 (17.3%) patients who met criteria for severe COVID-19 (including 42 [13.2%] with solid organ transplantation, 94 [19.9%] with CLL, and 71 [17.4%] with plasma cell malignancies), it was not possible to determine whether disease was severe at initial evaluation or whether mild-to-moderate disease later progressed to severe disease. Thirty (2.5%) patients died within 28 days of the positive SARS-CoV-2 test result.

**TABLE 1:** Demographic and clinical characteristics of COVID-19–vaccinated veterans with selected immunosuppressive conditions and mild-to-moderate or asymptomatic COVID-19* — Veterans Health Administration, United States, March–September 2022

Characteristic	No. (%)							
	Total		Solid organ transplantation		Chronic lymphocytic leukemia		Plasma cell malignancy	
	All cases N = 1,196	Reviewed^†^ n = 110	All cases N = 317	Reviewed^†^ n = 50	All cases N = 472	Reviewed^†^ n = 30	All cases N = 407	Reviewed^†^ n = 30
Age, yrs, mean (SD)	**71.6 (10.9)**	**70.5 (11.0)**	64.6 (10.5)	65.1 (10.9)	75.3 (9.7)	78.3 (9.2)	72.7 (9.9)	71.8 (7.2)
Male sex	**1,146 (95.8)**	**101 (91.8)**	294 (92.7)	45 (90.0)	457 (96.8)	26 (86.7)	395 (97.1)	30 (100)
**Race and ethnicity** ^§^								
Asian	**7 (0.6)**	**1 (0.9)**	5 (1.6)	1 (2.0)	1 (0.2)	0 (—)	1 (0.2)	0 (—)
Black or African American	**333 (27.8)**	**43 (39.1)**	109 (34.4)	19 (38.0)	69 (14.6)	9 (30.0)	155 (38.1)	15 (50.0)
Native American or Hawaiian	**15 (1.3)**	**1 (0.9)**	3 (0.9)	0 (—)	3 (0.6)	1 (3.3)	9 (2.2)	0 (—)
White	**766 (64.0)**	**61 (55.5)**	170 (53.6)	28 (56.0)	373 (79.0)	19 (63.3)	223 (54.8)	14 (46.7)
Hispanic or Latino	**91 (7.6)**	**11 (10.0)**	29 (9.1)	8 (16.0)	27 (5.7)	3 (10.0)	35 (8.6)	0 (—)
Unknown	**75 (6.3)**	**4 (3.6)**	30 (2.5)	2 (4.0)	26 (5.5)	1 (3.3)	19 (4.7)	1 (3.3)
**U.S. region where primary vaccination series was completed^¶^**								
Continental	**179 (15.0)**	**12 (10.9)**	44 (13.9)	5 (20.0)	69 (14.6)	2 (6.7)	66 (16.2)	5 (16.7)
Midwest	**202 (16.9)**	**16 (14.5)**	47 (14.8)	3 (6.0)	97 (20.6)	6 (20.0)	58 (14.3)	7 (23.3)
North Atlantic	**294 (24.6)**	**28 (25.5)**	86 (27.1)	13 (26.0)	110 (23.3)	10 (33.3)	98 (24.1)	5 (16.7)
Pacific	**228 (19.0)**	**21 (19.1)**	69 (21.8)	8 (16.0)	92 (19.5)	7 (23.3)	67 (16.5)	6 (20.0)
Southeast	**287 (24.0)**	**33 (30.0)**	70 (22.1)	21 (42.0)	103 (21.8)	5 (16.7)	114 (28.0)	7 (23.3)
Unknown	**6 (0.5)**	**0 (—)**	1 (0.3)	0 (—)	1 (0.2)	0 (—)	4 (1.0)	0 (—)
Vaccine booster**	**1,003 (83.9)**	**90 (81.8)**	272 (85.8)	40 (80.0)	384 (81.4)	22 (73.3)	347 (85.3)	28 (93.3)
**Comorbidity score (0–6),^††^ mean (SD)**	**1.3 (1.2)**	**1.2 (1.0)**	1.7 (1.0)	1.7 (0.7)	1.1 (1.2)	0.8 (1.0)	1.2 (1.2)	0.9 (1.0)
**Antiviral drug received** ^§§^	**335 (28.0)**	**0 (—)**	94 (29.7)	0 (—)	148 (31.4)	0 (—)	93 (22.9)	0 (—)
**Severe COVID-19** ^¶¶^	**207 (17.3)**	**12 (10.9)**	42 (13.2)	4 (8.0)	94 (19.9)	5 (16.7)	71 (17.4)	3 (10.0)
Death***	**30 (2.5)**	**2 (1.8)**	8 (2.5)	0 (—)	11 (2.3)	2 (6.7)	11 (2.7)	0 (—)

**Abbreviation:** VA = Veterans Health Administration.

* First documented SARS-CoV-2 infection after vaccination.

^†^ All patients in the reviewed subcohorts had mild-to-moderate COVID-19 or asymptomatic infection at the time of initial evaluation and were not given antiviral drugs.

^§^ VA patients self-report Hispanic or Latino (Hispanic) ethnicity separately from race; therefore, Hispanic patients are also included in the race categories. Persons of Hispanic origin might be of any race but are categorized as Hispanic; all racial groups are non-Hispanic.

^¶^ *Continental:* Arkansas, Colorado, Louisiana, Mississippi, Montana, Oklahoma, Texas, Utah, and Wyoming; *Midwest*: Illinois, Indiana, Michigan, Minnesota, Missouri, Nebraska, North Dakota, Ohio, South Dakota, and Wisconsin; *North Atlantic*: Connecticut, Delaware, District of Columbia, Maine, Maryland, Massachusetts, New Hampshire, New Jersey, New York, North Carolina, Pennsylvania, Rhode Island, Vermont, Virginia, and West Virginia; *Pacific:* Alaska, Arizona, California, Hawaii, Idaho, Nevada, New Mexico, Oregon, and Washington; and *Southeast*: Alabama, Florida, Georgia, Kentucky, Puerto Rico, South Carolina, and Tennessee.

** Receipt of ≥1 additional vaccine dose after completion of the initial vaccination series; no attempt was made to determine the number of additional doses.

^††^ Based on number of persons with the following six comorbidities: Alzheimer disease or other dementias, chronic kidney disease, chronic obstructive pulmonary disease, diabetes, heart failure, and peripheral vascular disease.

^§§^ Using VA pharmacy records, which do not detect prescriptions sent to non-VA pharmacies nor some medications given in the emergency department.

^¶¶^ Within 28 days of the positive results of SARS-CoV-2 test performed at the VA.

*** Defined as either death within 28 days of the positive results of SARS-CoV-2 test performed at the VA, or hospitalization with either administration of dexamethasone or evidence of hypoxemia (minimum pulse oximetry <94% or any use of supplemental oxygen).

### Reasons for Not Administering Antiviral Medications

Among 110 patients with mild-to-moderate COVID-19 at initial evaluation who did not receive antiviral drugs, 22 (20.0%) were offered treatment but declined (Table 2). Among the 88 who were not offered antiviral treatment, 20 (22.7% [18.2% of all 110 who did not receive antiviral drugs]) were not offered treatment because symptoms had been present for >5 days, and five (5.7% [4.5%]) were not offered treatment because of concern about possible drug interactions (in three cases, tacrolimus and ritonavir). In 63 cases (71.6% [57.3%]), no treatment was offered because the patient was considered asymptomatic (20 cases) or had mild disease (43 cases). In no cases was concern about Paxlovid rebound mentioned as a reason for patient or provider deciding against treatment. Sixteen of the asymptomatic infections were detected by hospital, emergency department, or transplant clinic screening procedures, or before elective procedures; 24 patients with mild illness received follow-up from clinical staff members only by telephone, with no documented reference to possible antiviral treatment.

**TABLE 2:** Reasons for nonreceipt of antiviral medication among COVID-19–vaccinated veterans with mild-to-moderate or asymptomatic COVID-19 and immunosuppressive conditions — Veterans Health Administration, United States, March–September 2022

Reason for nonreceipt of antiviral drug	Diagnosis, no. (%)			
	All N = 110	Solid organ transplantation n = 50	Plasma cell malignancy n = 30	CLL n = 30
**Antiviral offered (22)**				
Patient declined	**22 (20.0)**	13 (26.0)	7 (23.3)	2 (6.7)
**Antiviral not offered (88)**				
Symptom duration of >5 days	**20 (18.2)**	7 (14.0)	3 (10.0)	10 (33.3)
Concern about drug interaction*	**5 (4.5)**	3 (6.0)	0 (—)	2 (6.7)
Concern about Paxlovid rebound†	**0 (—)**	0 (—)	0 (—)	0 (—)
Patient asymptomatic§	**20 (18.2)**	10 (20.0)	6 (20.0)	4 (13.3)
Mild-to-moderate disease or reason not mentioned	**43 (39.1)**	17 (34.0)	14 (46.7)	12 (40.0)
Telephone follow-up only§	**24 (21.8)**	11 (22.0)	5 (16.7)	8 (26.7)

**Abbreviation:** CLL = chronic lymphocytic leukemia.

* The drug interactions noted were of ritonavir with tacrolimus for all three patients with solid organ transplantation. Among the patients with CLL, the interaction was ritonavir with a statin in one patient and not specified in the other patient.

^†^ A phenomenon in which symptoms recur after having resolved during a standard 5-day treatment with Paxlovid.

^§^ Description as asymptomatic or with the only follow-up of a positive SARS-CoV-2 test result by telephone are not mutually exclusive; among other cases, test results were obtained while the patient was present, either in the hospital, emergency department, or urgent care.

The study required review of 233 cases to obtain 110 that met inclusion criteria for analysis. The 123 excluded cases included 38 (16.3% of all 233 cases reviewed) in which chart review determined that an antiviral drug was given during mild-to-moderate COVID-19, although this had not been detected in the initial EMR screen. The undetected treatments included an outpatient regimen of remdesivir (23 patients), delivery of a monoclonal antibody at a VA (4 patients) or non-VA facility (1 patient), or delivery of an oral antiviral at a VA facility (4 patients) or non-VA pharmacy (6 patients).

## Discussion

During March–September 2022, among 110 patients in the VA system with solid organ transplantation, CLL, or plasma cell malignancies who had previously received a COVID-19 vaccine but who did not receive antiviral treatment after receiving positive SARS-CoV-2 test results during a mild-to-moderate or asymptomatic infection, 20% were offered treatment and declined. The remainder were not offered treatment because symptoms were present for >5 days (22.7% of those not offered treatment), concern about possible drug interactions (5.7%), or asymptomatic infection (22.7%), with no reason other than mild symptoms given for 43 of 88 (48.9%) cases.

Limited information is available on reasons for failure to prescribe antivirals to eligible patients with COVID-19. Algorithms to determine these reasons using EMR review would have to be based entirely on text data; therefore, measures to develop them would likely be prone to bias. VA EMR data also underestimate antiviral use, because chart review identified evidence of antiviral therapy in 16.3% of reviewed cases in which antiviral therapy was not detected through the initial EMR-based algorithm. Thus, reported use among 23.9% of the general VA population (*5*) and 28.0% seen in this study are both underestimates, but the proportion of eligible patients who are not offered an antiviral is still substantial.

### Limitations

The findings in this report are subject to at least six limitations. First, the study was conducted among a population of patients who were at particularly high risk for severe disease; drivers of limited antiviral use might be different among populations at lower, but still substantial, risk. Second, these results might not be applicable to non-VA health care systems. For example, results could differ in systems that do not routinely test all patients at hospital admission, that rely on offsite private pharmacies for prescriptions, that do not adhere to recommended timelines for antiviral treatment, or that have no standardized mechanisms for communicating with patients after positive test results. Rates of prescription of antiviral medications could be higher in other health care systems, but available data suggest prescription rates were similar in the VA and U.S. systems participating in PCORnet through mid-2022 (*5*,*6*). Third, results might not be generalizable to women or to persons of Hispanic, Asian, or Native American ethnicity and race, and who were underrepresented or not represented in this study. Fourth, EMRs underestimated antiviral use and have other limitations. However, focus on chart review ensured that all patients 1) had solid organ transplants, CLL, or plasma cell malignancies; 2) had SARS-CoV-2 infection; 3) were evaluated during mild-to-moderate COVID-19; and 4) did not receive an antiviral. Fifth, chart review, when it focuses on information not available through electronic searches, involves subjective judgment and limits the numbers of cases that can be assessed. Accordingly, a research question that could be answered with a small sample and would not require complicated analysis was selected. Finally, case review had to focus on clinical notes, which might not accurately reflect the conversations that occurred.

### Implications for Public Health Practice

Interventions that might improve prescription of antiviral medications for patients for whom they are indicated include 1) educating patients to inform health care providers soon after developing symptoms, especially if they have had previous discussions about antivirals and have decided they would be interested in receiving treatment; 2) educating providers, especially those tasked with making routine follow-up calls to patients to monitor their progress after a positive test result, about the indications for antiviral treatment to reduce risk for severe infection; and 3) ensuring that patients whose test results will return after they have left the facility have reliable contact information and a plan in place regarding antiviral treatment.

## Footnotes

*
https://www.fda.gov/media/155055/download

†
https://www.va.gov/vdl/documents/Clinical/Joint_Longitudinal_Viewer_(JLV)/jlv_2_9_ug.pdf

§ Care at a non-VA facility is not always documented in patients’ records. Although some patients might have received care at a non-VA facility, all 110 patients included in the analysis had sufficient VA data to be classified. Patients whose records suggested that they might have received an antiviral elsewhere were excluded from the analysis.

¶
https://www2.ccwdata.org/web/guest/home/

** Comorbidities included Alzheimer disease and other forms of dementia, chronic kidney disease, chronic obstructive pulmonary disease or bronchiectasis, diabetes, heart failure, and peripheral vascular disease.

†† 38 C.F.R 16.104d(4)iii.
